# Practical Guidelines for Implementing Longitudinal Faculty Development Programs

**DOI:** 10.15694/mep.2021.000101.1

**Published:** 2021-04-27

**Authors:** Jeannine Nonaillada

**Affiliations:** 1 NYU Long Island School of Medicine

**Keywords:** faculty development, longitudinal faculty development, adult learning.

## Abstract

This article was migrated. The article was marked as recommended.

Health professions faculty are often required to teach and participate in research efforts, although many have not undergone prescribed training in these areas. As a result, faculty development programs at medical schools and teaching hospitals typically offer single workshops or lectures formally addressing these subjects. Since longitudinal faculty development programs are known to be superior in fostering reflection and self-directed learning, these practical guidelines are provided for how to successfully implement such a program and sustain enrollment of participants over time. These guidelines are based on best evidence of faculty development models, personal experience of the author and grounded in theoretical frameworks about adult learning.

## Introduction

Faculty development has become an essential part of medical education culture, and is noted as being pivotal for institutions in order to promote academic distinction among their faculty ( Steinert, 2000). Although physicians and health professionals engage in teaching and participate in research efforts, many have not undergone formal training in these areas ( McCoy *et al.,* 2018, Wilkerson and Irby, 1998). Hence, faculty development programs focused on teaching abilities and research skills improvement can make valuable contributions and enhancements to the careers and professional growth of physicians. In order to keep such programs successful and sustainable, educators and administrators must weigh the balance of keeping things appealing to faculty, while offering formats that are convenient for full participation and commitment.

## Method

**Figure 1.**
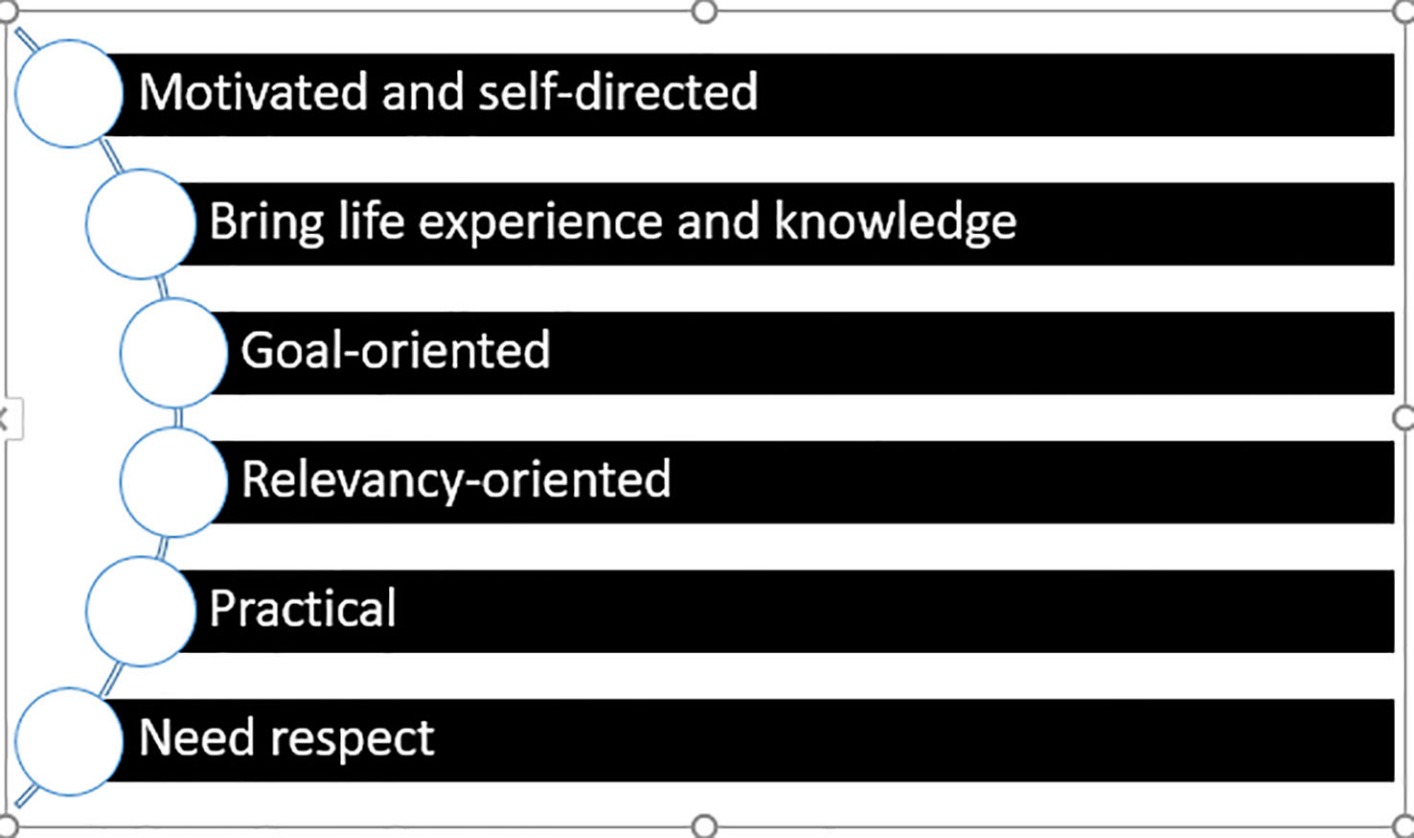
Characteristics of Adult Learners

The purpose of this article is to disseminate strategies to design and carry out longitudinal faculty development programs, meaning those that faculty continually participate in and commit to over an extended period of time. It is known that in contrast to one hour workshops or one-day retreats, longitudinal faculty development programs are beneficial since they allow time for reflection, feedback, and fostering self-directed learning ( Knight *et al.,* 2005). These practical guidelines frame faculty development within the context of offering formal longitudinal programs inclusive predominately of skills-building in teaching and research, with a target audience of primarily physician faculty in undergraduate and graduate medical education programs. However, these practical guidelines can likely be applicable to many other health professionals with formal development programs, including nurses, pharmacists, physician assistants, and rehabilitation therapists. The content of this article is meant to provide basic, generalizable guidance for medical schools and teaching hospitals looking to initiate such programs for their faculty. Guidelines are based on: personal experience from the author who is the Director of a longitudinal faculty development program that has been in inception since 2012 at NYU Long Island School of Medicine, and the underpinnings of adult learning theory ( Knowles, 2014) which the author has depicted in her own diagram based on this work Figure 1.

### Guideline 1

#### Secure Executive Sponsorship

It is crucial to ensure you have someone in a position of maximum influence to advocate for your program. Whether it be from the medical school Dean’s office or the medical center’s C-suite, the impact that is made from individuals holding executive leadership roles petitioning for your faculty development program is far reaching. Such individuals ensure equal access and availability to your program for all potential participants. With their operational clout, they also help to mitigate administrative barriers that may occur regarding budgetary or logistical issues that in contrast, individuals holding educational leadership roles may not have much influence over ( Kloppenborg, 2009). In order to secure executive sponsorship for your longitudinal faculty development program, you need to seek out potential sponsors early on before program roll-out, and include these individuals in the decision-making processes for your program.

### Guideline 2

#### Obtain Leadership Buy-In

The support of departmental leaders in your institution is vital for initial enrollment of participants in your longitudinal faculty development program, as well as for sustained involvement. If your program participants primarily hold clinical duties, then they will undoubtedly need the encouragement of their departmental chairs or division chiefs to protect the time they must devote to your longitudinal faculty development program. This is most successfully done by clearly laying out the time commitment and required deliverables for your program at the onset of participant recruitment. It is also beneficial to openly relay to departmental leadership the benefits that program participation has to their single clinical practices. For example, a participant’s deliverable may be to complete a departmental teaching plan encompassing new curricula for a specific residency program. Or, it may be to complete a research project whose outcomes will directly impact clinical decisions in practice. Both of these scenarios would be of high interest to departmental chairs and/or division chiefs looking to provide and promote best practice in teaching and research.

### Guideline 3

#### Establish Core Teaching Faculty

Like most large scale institutional efforts, it is important to acknowledge that it cannot be done by one single person. This is where you want to enlist the efforts of your most dedicated faculty to take on a curricular component of your longitudinal faculty development program. By having not one, but various faculty members teaching in your program, you expose participants to a variety of teaching methods and styles. It also provides teaching faculty with a sense of ownership to your longitudinal faculty development program, where they are contributing to its content and through the extension of the output of the participants, its overall impact. Just as with guideline two, you must be transparent with time expectations of your program’s teaching faculty, and give specific information about what type of teaching materials are involved and how they will be required to deliver them to program administrators. Provide timely feedback to your core teaching faculty after each session, as this gives them a solid basis from which to continually grow as senior educators of your institution. With each program cohort, there may be a need to make adjustments to your teaching faculty roster. Do not feel compelled to keep the same faculty teaching for fear of hurt feelings. Your goal is to deliver the best faculty development program, and to do so, you need to ensure those that are teaching are able to give their time and are the most qualified and effective faculty to teach in your program.

### Guideline 4

#### Offer Curricular Choices to Participants

As Malcolm Knowles’ theory indicates, the needs of adult learners vary as each person brings with them different life experiences and viewpoints ( Knowles, 2014). Thus, when building a longitudinal faculty development program, offering choices to participants is favorable ( Steinert, 2000). Perhaps some participants are more interested in development topics about teaching and learning, where other participants are more eager to learn about research or leadership. By designing various program pathways within your longitudinal faculty development program, participants can then opt for what is most appealing to them, and then, as adult learning theory suggests, they will be more successful in meeting your program outcomes as a result of selecting the curriculum they originally desired. Thus, constructing detailed syllabi with course readings and assignments for each curricular pathway in your longitudinal faculty development program will illustrate the substance of all available options for participants. Although this may be more labor intensive on the part of program administrators, the upshot of having participant satisfaction and productivity will make this worthwhile.

### Guideline 5

#### Implement Policies and Procedures for Structure

Although faculty development programs may be a means of recreation for clinical faculty to escape their day to day routines for patient care or working in the lab, there must be structure in the way they are conducted, especially if attendance is required at all classes/events. Program administrators need to decide what the attendance policy will be, if makeup classes or assignments will be offered if participants are absent, and how they will respond when procedural compliance issues arise among program participants. One possibility to ensure attendance at all classes/events is to have program applicants submit a signed letter of agreement that is co-signed by their department chair or division chief, acknowledging the requirement to be present at all classes/events to remain in the program. In order for this to be practical, it is best if classes/events in your longitudinal faculty development program are offered at the same time and day when at all possible. This allows for consistency and planning among program participants, as well as for your teaching faculty. Procedures for submitting any pre-work or required assignments and any deadlines should also be clearly stated in a shared document outlining your longitudinal faculty development program.

### Guideline 6

#### Incorporate Virtual Curricular Delivery

While face to face classes may be viewed as more traditional and for some, their preferred format for faculty development programs, the utilization of virtual delivery options for faculty development has certainly increased, especially with the COVID-19 pandemic ( Forde, 2020). Although having in-person classes allows your program participants with greater options to converse and cultivate personal relationships with fellow faculty, the use of virtual delivery to either complement or enhance your longitudinal faculty development program can be an efficient and cost-effective way to incorporate varied active teaching methods. For example, common teaching strategies such as the use of breakout rooms, real-time polling, and whiteboards can all be implemented virtually, without the use of other physical space or extra audiovisual equipment. Additionally, with virtual classes, faculty have greater flexibility to participate in a manner that accommodates their busy schedules: some may be finishing up in their patient clinic and can log into the classes conveniently from their office practice, while others may have childcare needs that require them to be physically home while they sign-in to class. Regardless of the individual circumstances, program administrators must acknowledge that program participants will be more appreciative and amendable to mandatory synchronous classes if they are given the freedom to attend from a location that best suits their current needs. To ensure not only the attendance of your participants, but also their engagement, it is wise to clearly make it known in advance what the expectations are for having cameras turned on for video representation and the preferred mode of participation, whether it be unmuting all microphones or using the chat feature to type in responses to questions.

### Guideline 7

#### Foster Communication among Program Participants

Building on guideline six, one tool that can be used to foster communication between your program participants are virtual discussion boards. Most electronic learning platforms have the option of enabling discussion boards where longitudinal faculty development program administrators can post a topic of interest or pose a question to program participants to elicit conversation or healthy debate surrounding a particular theme. This can be done as a pre-class virtual discussion to gauge how program participants feel in relation to the subject, or this can be done as a means to encourage participants’ reflection after the class. In turn, the program administrator must make it a point to respond timely to the posts on the discussion board, and to monitor individual contribution levels among each program participant.

### Guideline 8

#### Connect the Curricular Dots

Just as it is done within medical education curricular programs, the use of the spiral curriculum ( Harden, 1999) is a useful way to connect the dots in longitudinal faculty development programs. Your program syllabus should be designed with the end result in mind, meaning you should visualize what you want your program participants to achieve and how you want them to perform as faculty development program graduates. In doing this, they will be most successful in mastering your program content if they are able to cohesively make connections from one session to the next. To facilitate this among your teaching faculty, be sure to distribute the program syllabus beforehand, and offer suggestions to faculty on how they can have participants reflect on the prior session and weave it into the next session. For example, how to use learning theories (one class) as the underpinnings of developing your research study question (later class). Or, how to conduct small group teaching sessions (one class) while incorporating innovative technology in your lessons (another class). By connecting the dots, your program participants will be at less risk for compartmentalizing the wide range of content that they are receiving in your longitudinal faculty development program, but rather have a greater ability to become proficient in all aspects of the curriculum.

### Guideline 9

#### Implement Continuous Quality Improvement

As faculty developers often advocate for ongoing reflection of learners participating in educational activities ( Clayton and Ash, 2005), faculty development administrators need to regularly consider how they may improve their program to offer the best path for growth. This entails reflecting on both the educational design and delivery of curricula in your longitudinal faculty development program. Perhaps certain topics listed on your syllabi need to be revised, or even eliminated to make room for more timely and necessary topics as the needs of your institution evolve. This is natural and to be expected. Furthermore, the structure of your program may need to be changed. For example, as mentioned in guidelines four and six, varying the delivery methods to include virtual and face to face classes, or creating focused pathways that faculty can choose from. Continuous quality improvement of your longitudinal faculty development program also includes evaluating its impact. While you can have program participants self-rate themselves on their knowledge retention and skill acquisition, this manner of self-reported data has limitations. An alternative to this may be to examine the output from your program participants. For example, have new curricula been created by program graduates that is now being implemented in undergraduate or graduate medical education programs? Or, have new research studies been completed and published in peer-reviewed journals for wide dissemination? These are objective data points that can be used to display the impact and success of your program, and from which to gauge future program revisions that need to be made, and are useful to convey the value of your program to executive leadership at your institution as well.

### Guideline 10

#### Promote the Program

Take advantage of the many opportunities available to promote your longitudinal faculty development program both within your institution and outside of it. For increased internal program awareness, you can highlight your program’s curriculum or graduate achievements at institutional events, newsletters, or town hall meetings. Externally, you can submit an abstract to a national forum or meeting that may offer a specific category for faculty development, where you can disseminate your program’s success to a wider, diverse audience. This is also a great way to network with other faculty development program administrators, and meet new faculty who you can also then recruit for collaboration in your own program. It is also wise that your longitudinal faculty development program has a branded identity that can easily be identified with all marketing materials. For example, give your program a catchy name and display this name alongside your institution’s approved logo. You also may want to have a separate webpage for your longitudinal faculty development program within your institution’s website that faculty can utilize to learn about the program and its key features.

### Guideline 11

#### Give Public Recognition

Be sure to give public recognition at widely-attended events in your institution where you can express appreciation for your longitudinal faculty development program participants. As busy clinicians/scientists, faculty will be grateful that their time and efforts are publicly recognized within your institution, especially if they do not receive protected time to engage in faculty development activities. As a result, their determination to succeed in your program may increase once they have confirmation that their work is being acknowledged among their senior leaders and peers. Examples of venues for recognition can be annual faculty meetings or research showcases. Congruently, give communal appreciation to your teaching faculty for their involvement in your program.

### Guideline 12

#### Show Post Graduation Prospects

Faculty may be motivated to enroll in your longitudinal development program if they can clearly see a connection between their participation and the trajectory of career prospects that may take place afterwards. Perhaps your program provides them with increased skills to design a new undergraduate course, with leadership skills necessary to take on the role of residency program director, or the foundations to apply for federal grant funding for a new clinical trial. By showing faculty the linkage between your longitudinal development program and where it may take them, you are providing them with a well-defined path that they may not have envisioned on their own for themselves. Another way to accomplish this is to have past graduates speak at information sessions regarding your longitudinal faculty development program, where they can share with potential applicants their experiences going through the program and how it has enhanced their career as faculty.

## Conclusion

Longitudinal faculty development programs offer a way towards professional growth for physicians and other health professionals. In order to ensure vitality of these programs, key components include offering participants with choices in curricula, providing clear expectations, and demonstrating attainable pathways from the program to career enhancement. Partnerships between faculty development program administrators with executive sponsors and institution leadership are also instrumental in achieving effective longitudinal faculty development programs. See Table 1 for a checklist to aide in creating longitudinal faculty development programs. Overall, constructing an accomplished longitudinal faculty development program is a time-consuming, demanding, and multi-faceted task, but the efforts are reinforced with the progress of all those involved.

**Table 1.** Checklist for Implementing Longitudinal Faculty Development Programs

Questions to Consider:	Solutions to Explore
1. Who is our executive sponsor?	
2. Have we appealed to leadership?	
3. Who is our core teaching faculty?	
4. Are choices offered in the curriculum?	
5. Are there clear program policies?	
6. Is there virtual participation?	
7. How will participants communicate with each other outside of sessions?	
8. Is the curriculum related from session to session?	
9. How will continuous quality improvement be performed?	
10. How and where will we promote the program?	
11. How and where will we recognize the achievements of program participants?	
12. How and where will we highlight the potential opportunities and future roles for program graduates?	

## Take Home Messages

• Longitudinal faculty development programs are superior in fostering reflection and self-directed learning.
• By incorporating principles of adult learning into program curricula, longitudinal faculty development programs can elicit participant motivation, and lead to faculty’s career trajectory of professional growth.
• Implementing a longitudinal faculty development program is a demanding and time-consuming undertaking that requires institutional support for optimal success.

## Notes On Contributors

Dr. Jeannine Nonaillada is Assistant Dean of Faculty Development and Mentoring at NYU Long Island School of Medicine, New York. She is an Associate Professor in both the Department of Medicine and the Department of Rehabilitation Medicine. Dr. Nonaillada has been a licensed occupational therapist for twenty years. ORCiD: https://orcid.org/0000-0002-7424-0774
