# Supplementary material for: Practical Guidelines for Implementing Longitudinal Faculty Development Programs
Source: MedEdPublish (2016). 2021 Apr 27;10:101. [Version 1] doi: 10.15694/mep.2021.000101.1 (PMC10939650; doi:10.15694/mep.2021.000101.1)
Supplement: Peer review history

# Reviewer report: Reviewer response for version 1

**DOI:** 10.21956/mep.19099.r27532

**Published:** 2021-05-23

**Authors:** Helena Filipe

**Affiliations:** 1 Hospital of the Armed Forces/PL-EMGFA

This review has been migrated. The reviewer awarded 4 stars out of 5 Thank you for the opportunity to review this article.The Author creates the case to underpin and justify the benefits of the longitudinal learning format applied to faculty development. Weaving learning experiences into daily practice in seamless continuity and synergy with learners’ interests nurtures an engaging learning environment workplace.A word on Margolis et al. work on the “extended congress” would come in tandem and further support the “extension of a scientific meeting into a framework where learning can be integrated with the demands of work/life” (1). As highlighted in the article, technology may assist to reach a wider and diversified audience in a learning format that extends learning opportunities in time and space including in the participants' practice setting. Combining evidence and experience, the Author describes key guidelines to be successful in establishing a longitudinal faculty development program, explaining their importance, and providing useful suggestions to the reader.Well sustained on adult learning principles, guideline 4 especially sparked my interest by highlighting the potential numerous learning opportunities, the participant might be offered to select based on personal preferences. “Single workshops or lectures “can be transformed into longitudinal, spaced learning experiences to promote self-direct learning, elicit personal reflection, and enable dissemination and reinforcement of new learning at the workplace. The importance of involving several stakeholders and the need of the organisations’ support is underlined.The table can work as a practical tool for a systematic approach in practice. This paper is useful for the medical education community especially interested in curriculum development and education management, to design, implement, pilot, and iteratively enhance or adapt faculty development programs into the longitudinal format delivery.Reference1. Margolis A, Balmer J, Zimmerman A, López-Arredondo A, 2020, 'The Extended Congress: Reimagining scientific meetings after the COVID-19 pandemic', MedEdPublish, 9, [1], 128, https://doi.org/10.15694/mep.2020.000128.1

Reviewer Expertise:

NA

No decision status is available

# Reviewer report: Reviewer response for version 1

**DOI:** 10.21956/mep.19099.r27533

**Published:** 2021-05-20

**Authors:** Hui Meng Er

**Affiliations:** 1 International Medical University

This review has been migrated. The reviewer awarded 4 stars out of 5 The author has provided insightful tips for implementation of faculty development programs. The checklist in Table 1 is very useful to guide the program team to think about the strategies to promote and ensure sustainability of the programs in relation to the context of the institution. Faculty needs analysis is another important exercise to determine the gaps in the competencies among the faculty in the institution and guide the curriculum design of the faculty development programs. This not only helps the institution to manage the resources more efficiently, but also provides a strong rationale to encourage participation.

Reviewer Expertise:

NA

No decision status is available

# Reviewer report: Reviewer response for version 1

**DOI:** 10.21956/mep.19099.r27531

**Published:** 2021-05-03

**Authors:** Sarmishtha Ghosh

**Affiliations:** 1 International Medical University

This review has been migrated. The reviewer awarded 4 stars out of 5 Despite realizing the importance of faculty development activities in educational institutions, what is most often underrated is the evaluation of those programs to check the utility and importance in the production of a trained workforce in the organization.FDAs involve a lot of resources and hence it is important that it is properly evaluated to show changes in the behavior of the faculty. Factors like unplanned implementation, personal choices, or just pretest -immediate post-test are used to indicate the effectiveness of the FDA. This article is useful as it refers to the development of reflective ability and also self-directed learning using adult learning principles. The structured guidelines will be helpful in drafting the FDAs in any organization, making them more contextual and effective.

Reviewer Expertise:

NA

No decision status is available

# Reviewer report: Reviewer response for version 1

**DOI:** 10.21956/mep.19099.r27534

**Published:** 2021-04-28

**Authors:** Mark Di Corcia

This review has been migrated. The reviewer awarded 5 stars out of 5 Despite the fact that it is an LCME requirement and all schools would say that their institutions provide "faculty development" it is the rarer instance whereby it is implemented from an identified theoretical framework or a longitudinal programmatic design. The problem is compounded when attempting to develop busy faculty members including those with clinical duties in addition to teaching/education. Therefore, having a set of pragmatic guidelines and a checklist as those outlined in this article can be invaluable when attempting to strategize, design, allocate resources, implement, and track outcomes for a program of required (and needed) faculty development especially within the confines of our ever increasing resource constrained environments.

Reviewer Expertise:

NA

No decision status is available
